# Human Brain Organoids in Migraine Research: Pathogenesis and Drug Development

**DOI:** 10.3390/ijms24043113

**Published:** 2023-02-04

**Authors:** Parisa Gazerani

**Affiliations:** 1 Department of Life Sciences and Health, Faculty of Health Sciences, Oslo Metropolitan University, 0130 Oslo, Norway; parisaga@oslomet.no or gazerani@hst.aau.dk; 2 Centre for Intelligent Musculoskeletal Health (CIM), Faculty of Health Sciences, Oslo Metropolitan University, 0130 Oslo, Norway; 3 Department of Health Science and Technology, Faculty of Medicine, Aalborg University, 9220 Aalborg East, Denmark

**Keywords:** migraine, headache, brain, organoids, brain organoid, human brain organoids, neurological, psychological, pathogenesis, drug development

## Abstract

Human organoids are small, self-organized, three-dimensional (3D) tissue cultures that have started to revolutionize medical science in terms of understanding disease, testing pharmacologically active compounds, and offering novel ways to treat disease. Organoids of the liver, kidney, intestine, lung, and brain have been developed in recent years. Human brain organoids are used for understanding pathogenesis and investigating therapeutic options for neurodevelopmental, neuropsychiatric, neurodegenerative, and neurological disorders. Theoretically, several brain disorders can be modeled with the aid of human brain organoids, and hence the potential exists for understanding migraine pathogenesis and its treatment with the aid of brain organoids. Migraine is considered a brain disorder with neurological and non-neurological abnormalities and symptoms. Both genetic and environmental factors play essential roles in migraine pathogenesis and its clinical manifestations. Several types of migraines are classified, for example, migraines with and without aura, and human brain organoids can be developed from patients with these types of migraines to study genetic factors (e.g., channelopathy in calcium channels) and environmental stressors (e.g., chemical and mechanical). In these models, drug candidates for therapeutic purposes can also be tested. Here, the potential and limitations of human brain organoids for studying migraine pathogenesis and its treatment are communicated to generate motivation and stimulate curiosity for further research. This must, however, be considered alongside the complexity of the concept of brain organoids and the neuroethical aspects of the topic. Interested researchers are invited to join the network for protocol development and testing the hypothesis presented here.

## 1. Human Organoids

**Figure 1:**
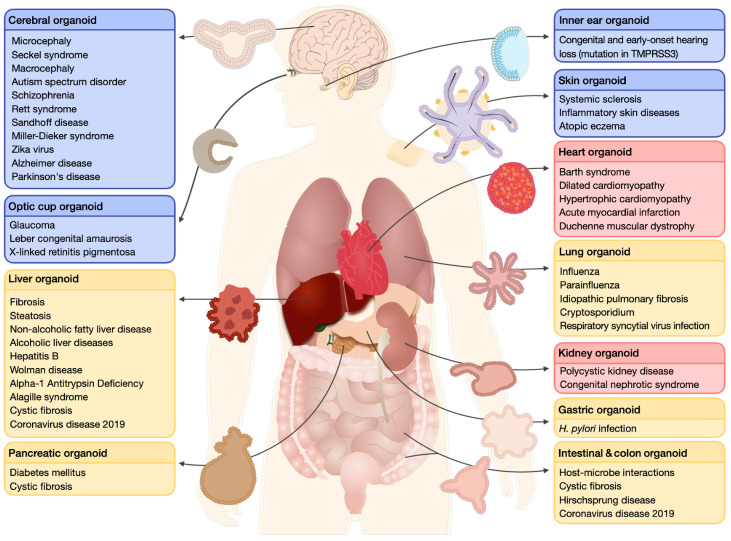
Examples of human organoids and examples of their applications in various disease models. This figure has been reproduced with permission from [10], granted by the Copyright Clearance Center’s RightsLink^®^ service (License Number: 5455920726198).

Human organoids are in vitro cultured three-dimensional (3D) structures that are mainly produced from two types of cells: (1) human pluripotent stem cells (hPSCs) that include both embryonic stem cells (ESCs) and iPSCs, and (2) organ-specific adult stem cells (ASCs or AdSCs). They are derived from individuals and recapitulate the cellular heterogeneity, structure, and functions of original human organs [1,2]. As organoids carry the genetic information of the source, they hold great potential for biomedical research and preclinical drug testing [3]. In the future, it is expected that human organoids will become one of the major sources of personalized regenerative medicine, gene repair, and transplantation therapy [4,5]. In theory, human organoids can be created from any organ and used in both basic and clinical research [2]. Researchers have succeeded in generating various types of organoids [2,3] with the aid of well-defined protocols and advanced technologies for in vitro differentiation [6,7]. For instance, retinal, kidney, liver, lung, gastrointestinal, cardiac, and brain organoids have been created [8,9] for disease modeling, drug screening, and regenerative therapy [10] (Figure 1).

### Disease Modeling and Drug Screening: From Animal Models to Human Organoids

Disease modeling and drug screening have long been carried out with the aid of various methods. Animal models, for example, have been successfully used in biomedical research for more than 100 years and could improve our knowledge of cellular and molecular signaling pathways, identification, and testing of potential drug targets, in addition to clarifying underlying pathological and disease mechanisms [11]. However, differences between animals and humans, and the lack of translation of findings from animal models to humans in a great deal of research, have become major obstacles to the application of animal models in disease understanding and drug discoveries [12,13]. Animal models have therefore been subjected to replacement strategies on the grounds of these limitations, along with ethical and welfare concerns, and the general recommendations of the 3Rs (replacement, reduction, and refinement) [14]. 

To reduce the translational gap [15], human in vitro biological systems [16] have emerged and been optimized over time. Conventional two-dimensional (2D) cell culture systems have been and continue to be used because they are less expensive, well-established, have possibilities for comparative literature, and allow for easier cell observation and measurement [17]. Tissue stem cell culture systems of hPSC and AdSC have provided an in vitro platform of human cellular materials for studying health and disease and testing pharmacologically active compounds or large-scale drug screening [2,18]. The first human embryonic stem cells (ESCs) line was introduced in 1998 [19]. Human iPSC (hiPSC) technology, established in 2007, is now widely used to generate “disease-in-a-dish” models [8,20,21]. This technology enabled disease modeling toward precision medicine [21]. The development of clustered, regulatory, interspaced, short, palindromic repeat (CRISPR)/Cas9 endonucleases has enabled the creation of genetically edited hiPSC-based disease models [22,23]. However, iPSC-derived 2D cell cultures have some limitations compared with 3D cell cultures [24]. 2D cell cultures have some limitations, for example, lack of a hierarchical structure, dimensionality, and cellular diversity. In some cases, cell-to-cell or cell-to-matrix interactions are limited in 2D cultures, which can influence certain cellular functions [2,25,26]. 

With the advancement in technology, 3D cultures have received high interest because of their more organ-like structures and the potential for the simultaneous culturing of different cell types [27,28] that can better mimic physiologically relevant organ systems [29]. By modifying the combination of growth factors and cell isolation procedures, researchers could generate multiple kinds of human organoids [30]. Improvements in protocols for the development of various organoids have resulted in a prolonged maturation time [31]. In addition, the generation of assembloids could allow a combination of more organoids to co-exist and better resemble the complexity of organs [32,33]. The term “assembloid” was first described by Dr. Sergiu Pașca as a 3D structure formed from the fusion and functional integration of multiple cell types which mimics the complex cellular interactions that exist in the body organs [34]. In such a system, both genetics and epigenetics [35,36] can be investigated. In addition, the transplantation of organoids into animals such as rodents has been reported, for example, the successful transplantation of whole-brain organoids into the adult mouse brain has been presented [37], where the researchers demonstrated evidence for anatomical and functional integration. The transplanting strategy somewhat resembles similar ideas of grafting structured neural tissues for stem-cell-based treatments that have already been carried out in human clinical trials, for example, in stroke, traumatic brain injury, and Parkinson’s disease [38].

**Figure 2:**
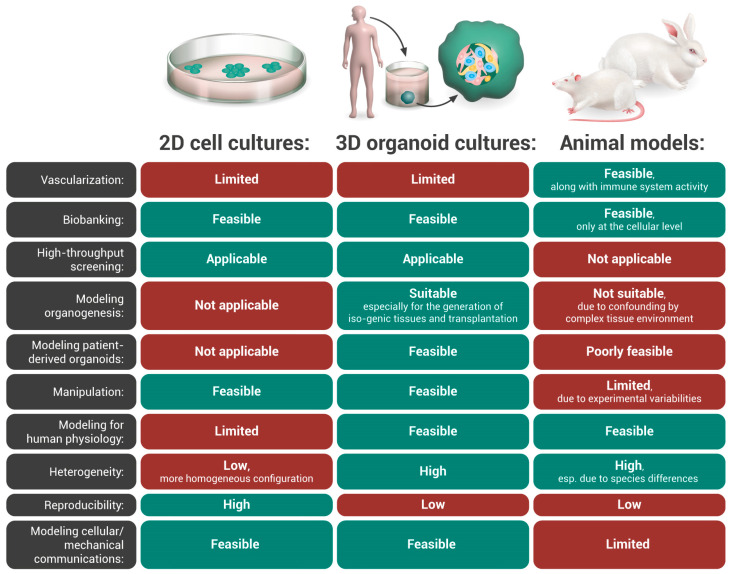
Potential and limitations of 2D cell culture systems, 3D organoid cultures, and organoid transplantation in animal models. This figure has been reproduced according to [10], with permission granted by the Copyright Clearance Center’s RightsLink^®^ service (License Number: 5455920726198).

Figure 2 depicts the potential and limitations of 2D cell culture systems, 3D organoid cultures, and the establishment of organoids in animal models. It is important to emphasize that no model is perfect [39]. Thus, the selection of a model must be based on the research question, and the conclusions must still be viewed cautiously.

## 2. Human Brain Organoids

The brain is one of the most complex and advanced organs in the body [40]. Brain complexity [40] has often limited easy access or thorough investigation of the brain in health and disease. Knowledge of the human brain has mostly been obtained from the investigation of postmortem brain samples, and now, with the advancement of human stem cells [8,41], brain regions can be produced. For example, the cerebral cortex can be produced from progenitor cell populations that organize and produce mature cortical neurons. Such cerebral organoids [42] have been shown to recapitulate features of human cortical development and allow the study of the regional developmental stages of the brain. On the other hand, stem cells originating from patients could help in modeling disorders [43], for example, microcephaly [44]. Interestingly, this disorder cannot be modeled in animals (unsuccessful models in mice); therefore, using organoids [45] could provide the potential for studying this disorder [46].

In general, neuronal differentiation and developmental problems can be studied in patient organoids to empower scientists in explaining disease phenotypes. Diverse and advanced technologies can be coupled with brain organoids [47], for example, in genome editing, single-cell sequencing, biomaterials, and bioengineering to embed and supply the brain–blood barrier, vasculature [48], and non-neuronal cells (e.g., microglia) [49].

A consensus in 2022 has been introduced on the nomenclature for nervous system organoids and assembloids to harmonize naming across users and for ease of understanding and applications in the field [50]. Attempts have also been made to organize a platform for sharing protocols where researchers can exchange, revise, and optimize protocols for use in the brain organoid field [51].

### Human Brain Organoids for Neurological Disorders

According to the latest global burden of diseases, neurological disorders are among the leading causes of death and disability worldwide [52,53]. Neurological disorders consist of a heterogeneous group of conditions, broadly characterized by peripheral and/or central nervous system deficits, issues, and malfunctioning. Although the etiology of neurological diseases varies greatly, they share some characteristics, such as heterogeneity of clinical presentation and diversity of cellular and molecular pathways [54]. Currently, most neurological disorders remain poorly treated due to a lack of efficient therapies. The latter is rooted partially in a poor understanding of the etiology of these disorders that originates from a lack of translation from basic to clinical modeling of disorders and/or drug testing.

**Figure 3:**
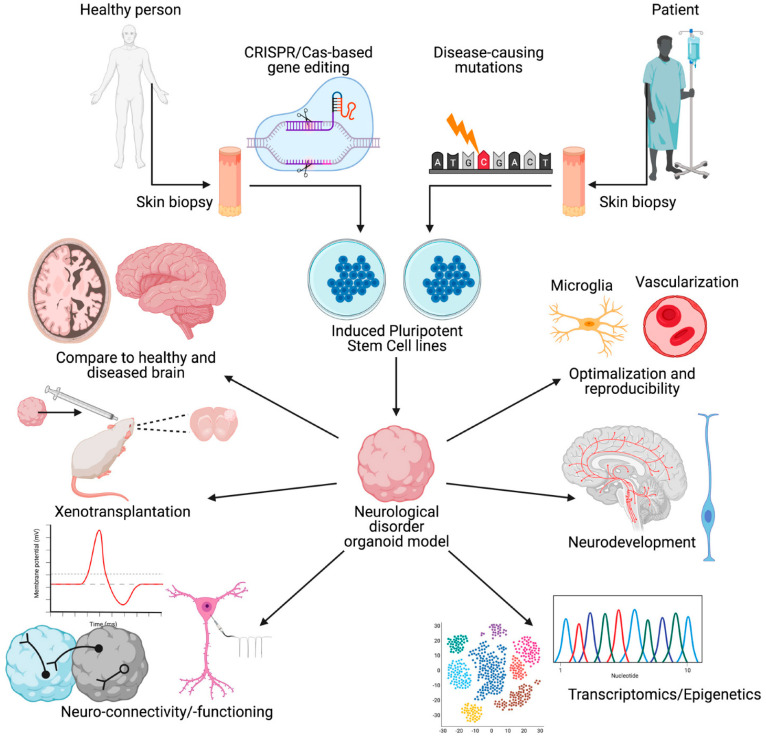
Schematic overview of the creation of and uses for organoid models of neurological disorders. Neurodegenerative, neurodevelopmental, and neuropsychiatric disorders can be modeled with the aid of brain organoids. Both neurofunctional and neurodevelopmental aspects can be investigated with a possibility for comparison between healthy and diseased brain organoids depending on the source or manipulations introduced, such as genetic manipulation and environmental stressors. In addition to the use of these models for studying pathogenesis, xenotransplantation of brain organoids into experimental animal brains has been proposed for treatment purposes or further studies in vivo. In these models, neural connectivity between different regions can also be studied, for example, in the assembloids of two brain regions. This figure has been reproduced from [60] under an open access Creative Common CC BY license granted by MDPI.

Human brain organoids provide a promising platform to recapitulate histological features of the human brain, model neurological disorders, and advance their treatments [3,55], for example, the development of organoids derived from patients’ brains can provide new insights into the mechanisms of a diverse range of neurological disorders, including neurodevelopmental and neurodegenerative disorders such as schizophrenia, epilepsy, Alzheimer’s disease, and Parkinson’s disease. Human brain organoids are not only used for understanding such disorders but can be used for drug screening and perhaps as a novel tool for treatment. As is seen in Figure 3, besides the application of CRISPR/Cas9 [56,57], it is expected that other new technologies such as high-throughput single-cell omics, gene editing, artificial intelligence, and machine learning could push forward precision medicine in neurological disorders when combined with brain organoid technology [58,59]. However, brain organoid technology is not yet at a level to completely mimic all of the interactions and structures of the human brain in vivo, and it is not clear whether it would be needed and for what purpose [60]. Meanwhile, assembloids have been applied to model interneuron migration and neuronal projections [61]. One interesting and important area is the neuroendocrine system and its role in various neurological disorders [33]. Researchers have speculated that brain organoids and assembloids are beneficial in modeling the development, regulation, and dysregulation of the stress system to understand a variety of stress-related neurological disorders [33].

## 3. Human Brain Organoids for Migraine

### 3.1. Migraine

**Figure 4:**
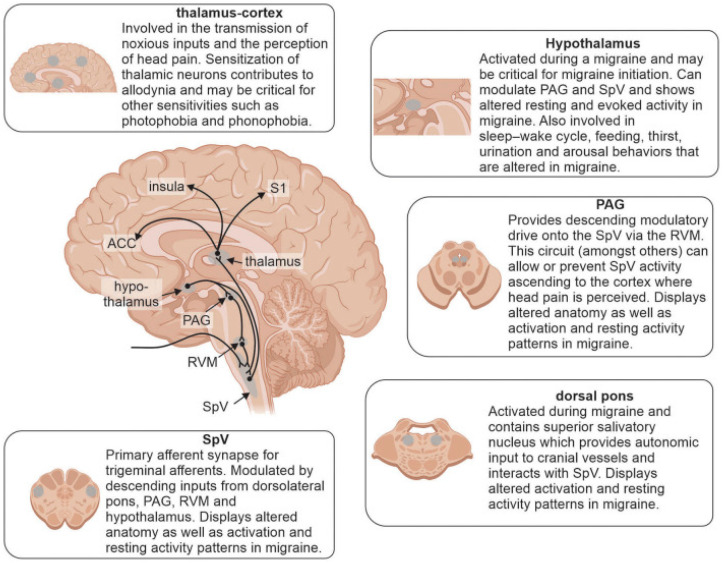
Brain regions involved in underlying mechanisms of migraines and reported alterations. Modulation of incoming noxious inputs: spinal trigeminal nucleus (SpV), periaqueductal gray matter (PAG), rostral ventromedial medulla (RVM), and dorsal pons. Higher order processing: hypothalamus, thalamus, anterior cingulate cortex (ACC), insula, and primary somatosensory cortex (S1). This figure has been reproduced from [72] under an open access Creative Common CC BY license granted by Frontiers.

Migraine is a prevalent neurological disorder that is often underestimated for its impact and consequences [62]. According to the global burden of disease, migraines are one of the top causes of disability worldwide [63]. It is estimated that migraines affect around 20% of people at some point in their lives [64]. They affect women more than men [65] and appear with re-occurring intense headaches and altered sensory and motor manifestations [66,67]. Attacks of migraines seem to follow an evolving phenomenon that occurs over time mirroring the phases that are each marked with certain symptoms as a result of certain neural mechanisms [68]. The mechanisms behind this evolutive process are only partially known, but neuroinflammation and central sensitization, influenced by genetics [69] and epigenetic factors [70], are proposed to play an important role [68,71,72,73,74]. Abnormalities in neuronal activity of several brain regions have been reported, for example at the spinal trigeminal nucleus, periaqueductal gray, rostral ventromedial medulla, and dorsal pons. Degrees of dysfunction have also been presented to occur in the hypothalamus, thalamus, anterior cingulate cortex, insula, and primary somatosensory cortex. Figure 4 depicts the regions in the brain that are suggested to contribute to the underlying mechanisms of migraines [72].

Migraine is a lifelong neurological disorder with an evolutive age-dependent feature in its prevalence and clinical manifestation [68]. Understanding migraine pathogenesis is challenging, however, progress has been made in genetics, functional, and anatomical changes accompanying migraines [75,76]. For example, genome-wide association studies (GWAS) using single nucleotide polymorphisms (SNPs) have shown several variants of genes that contribute to the neurological and vascular pathways in migraines [69]. The partial understanding of migraine pathogenesis has resulted in suboptimal treatment [77]. Hence, attempts continue to provide mechanism-based targeting of migraines with acute or preventive therapies [78,79]. One example is the involvement of CGRP in migraine pathogenesis and novel anti-migraine drugs developed to block CGRP or its receptors.

### 3.2. Modeling Migraine

Migraine is a complicated disorder proposed to appear as a result of a complicated web of interacting pathogenic alterations [80]. Due to this complexity, and species differences, many aspects of migraines cannot be modeled in animals [81,82]. Furthermore, the pathology and changes in disorder faced over time make it difficult to model accurately. Many in vivo animal models have been used to study certain aspects of migraine disorder but often have a shortcoming that might be related to environmental factors, genetics, or both [83]. Another limitation is that drug testing in animal models has often not proven successful because of the limited translation value [84].

Human experimental models of pain have been introduced and used for various purposes, including understanding pain and testing analgesics in the early stages of drug development [85]. Craniofacial and orofacial pain experimental models [86,87] have been used to assist in modeling aspects of migraines but remain incomplete due to their limited nature and ethical considerations. In human experimental pain models [88], various types of stimuli (e.g., chemical, mechanical, electrical) have caused short-lasting states of pain and sensitization that can be measured by various methods (e.g., quantitative sensory testing, brain imaging, biological biomarkers). The analgesic efficacy of drugs can also be tested using these models [89].

Human postmortem brain samples can provide tissues to be used for further analytical purposes. Such samples carry the genetic code, and if obtained from patients with disorders linked to genetic mutations, for example, migraines can be highly relevant for pathogenesis studies. However, access to high quantities of such materials is limited and most of the obtained tissues are aged and reflect an end stage of the disease; hence, it is impossible to evaluate the disease onset and developmental process. In addition, in vitro modeling of the obtained tissues may show abnormalities, for example, human dura mater cells obtained postmortem have been used for the establishment of a human in vitro model that has shown phenotypic, transcriptomic, and genetic abnormalities that must be taken into account for disease modeling [90].

Studying migraine as a neurological disorder in live humans is complicated due to unexpected attacks of headaches and access to patients for brain imaging during the attack [91]. In addition, limitations exist due to ethical and practical issues for provocation studies, where migraines can be experimentally induced by infusion or injection of triggering factors, such as nitroglycerin [92]. In addition, a migraine is not just a headache and can affect multiple other organs [93]. Therefore, studying migraines requires more than brain imaging alone. Consequently, researchers have taken a holistic approach to understanding the migraine phenomenon. Importantly, functional brain imaging coupled with other biomarkers could reveal aspects of migraine comorbidities [94], for example, functional neuroimaging studies have opened up possibilities to investigate the hypothalamus and the brainstem involved in the pathophysiology of a migraine. The thalamus may also contribute to the clinical manifestation of migraines [95] and abnormal thalamocortical network dynamics have presented in migraines [96]. Both functional and structural abnormalities have been demonstrated at the cortical level, mainly in the visual areas [94]. The current standard is to use a combined panel of biomarkers for studying migraine pathogenesis and response to treatments [97] to obtain a more complete picture of precision medicine for this disorder.

### 3.3. Can Human Brain Organoids Be Used as a Substitute Model for Migraine Disorder?

Considering the potential for the creation and application of human brain organoids, it is not irrational to consider that human brain organoids can also be applied to help in understanding the pathogenesis of migraines. The fundamental basis for this opinion is that migraine is a brain disorder that has been proposed to be linked to both genetic and epigenetic factors [70,80,98]. Hence, brain organoids can potentially be used for this purpose. Theoretically, both guided organoids (neural organoids resembling regions or domains of the nervous system) and unguided organoids (pluripotent stem cells differentiated in self-organizing 3D cultures) [50] can be explored for this purpose. Since brain imaging techniques have already demonstrated the connection between various brain regions [99], it is rational to consider assembloids [50] (for example a combination of different specialized cell types) in migraine research. Assembloids are also generated from a combination of two types of organoids (e.g., a fusion of dorsal with ventral forebrain) [50], and this can potentially offer a research value in modeling migraines.

A relevant benefit of an unguided brain organoid is that the organoids can survive in culture for approximately one year [60], permitting tests of various factors during the maturation of neurons and brain development that might be involved in the migraine-developing brain. Therefore, it has been speculated that brain organoids might offer a powerful tool to study migraine pathogenesis, drug screening, and even the use of brain organoids as a tool for treatment [100]. An optimal model of migraine must allow for studying the onset, development, pathology, and causes of the disorder accurately. In addition, an optimal model would provide the possibility to investigate malfunctioning pathways, abnormal protein interactions, and pathological mutations. Last, but not least, an optimal model would permit drug testing and identification of mechanism-based therapeutic effects. The latter is important because most of the available drugs for migraines are aimed at symptomatic therapy, not disease modification or cure of migraines [101]. Current brain organoids have limitations and shortcomings; therefore, for modeling migraines with the aid of brain organoids, the shortcomings of available models must be taken into account, as is explained in the below section.

## 4. Potential and Limitations of Brain Organoids for Migraine

Conceptualizing the theories behind migraine pathogenesis [102], for example, neurovascular theory [103], can help rationalize which brain organoids should be used and how they can be used for understanding migraines. For example, the neurovascular mechanisms of CSD (migraine aura) or neurovascular coupling and uncoupling in CSD are proposed to be changes with a longer-lasting effect than the initial CSD response and may be involved in the delayed activation of nociceptive pathways [104]. Aura development and cortical spreading depression (CSD) in migraines can potentially be studied further with the aid of brain organoids. Neuropeptide release evoked by KCl can serve as a quantitative measure of nociceptor excitation [105].

Human brain organoids can be used to study the impact of genetic mutations (e.g., channelopathy [106]) and environmental factors (e.g., environmental stress [107]) on the onset, development, and progression of migraines. Brain organoids in migraines can be used to investigate altered neuronal or metabolic pathways, protein, and mRNA expression, similar to what has been used for other neurodevelopmental or neurodegenerative disorders [60,108]. New single-cell proteomic techniques are powerful tools to identify protein function and alterations and provide valuable insights into post-translational modifications [109].

Several brain structures have been proposed to play a role in migraine pathogenesis including the trigeminal cervical complex (TCC) [110], periaqueductal gray (PAG), locus coeruleus (LC), rostral ventromedial medulla (RVM), thalamus, and hypothalamus. Human organoids of these regions can be considered relevant. The hippocampus is another important region. A potential link [111] between migraine and transient global amnesia (TGA) [112] has been proposed concerning anatomical and physiological disorders of the hippocampus, although controversies are present in the current literature. Migraines are often comorbid with several other brain disorders, and this feature opens up possibilities for studying commonly involved brain regions in migraine co-morbid conditions.

Brain organoids have also been used to study genetic and epigenetic regulation of the brain, neuronal connectivity, and neuro-immune interactions in healthy and diseased brains [60,113]. These are relevant aspects of migraine pathogenesis that must be considered in the development and use of brain organoids for migraines. In addition, iPSC-derived cell types can undergo genetic modifications using CRISPR/Cas9, as has also been proposed for clinical applications [114]. Furthermore, optogenetic tools can be introduced into the individual cellular components before implementation in an engineered organoid [115].

The iPSC-derived trigeminal ganglia (TG) organoids can be generated with iPSCs differentiating into sensory nociceptors, mechanoreceptors, and proprioceptors to study their actions in migraines. The usage of sensory ganglia organoids has previously been addressed for dorsal root ganglia (DRG) [116]. These components are considered a part of the PNS and hence can assist in understanding peripheral aspects of migraine pathogenesis.

Since no brain organoid exists for migraine, the speculation is to develop model(s) step by step and optimize them over time. The task is to ensure that the model would accurately resemble the disorder in vivo. Allowing for the development of a vascular system and the presence of microglia are important aspects to consider for balancing the ratios between neurons, and including glial cells for proper maturation, interconnectivity, and homeostasis within brain organoids [60,117]. At present, brain organoids face apoptosis in the core because the vascular system aids the circulation of nutrients, and gas exchange does not develop, hence waste removal is a challenge. This challenge has been noted in neurological disease modeling with organoids, for example, in stroke research [118]. Current attempts to vascularize organoids include the adjustment of culture protocols, the introduction of microfluidic devices [119], and the transplantation of organoids into highly vascular tissues in immunodeficient rodents [120]. Adjustment of protocols has been tried by the implementation of endothelial cells in the developing organoids to result in an elementary circulatory system [121]. IPSC-derived microglia have also been added to organoids to create a self-renewing microglia population [122].

The connections among distinct brain regions in migraine pathogenesis can potentially be reproduced in brain assembloids to assist in understanding alterations in the connectivity of neural networks in this disorder. Addressing connectivity in in vitro research might be challenging, but advancements in assembloids can generate a robust model to facilitate translating connectivity measurements that are recorded by neuronal imaging in human brains to possible investigations in human brain organoids [123]. Advances in bioengineering techniques will most likely help overcome the existing challenges in developing organoids [28] or assembloids [34] for migraines. In this vein, modeling human nociception, by reprogramming patient-derived iPSCs into organoids with relevant cell types, has also been proposed [105].

**Figure 5:**
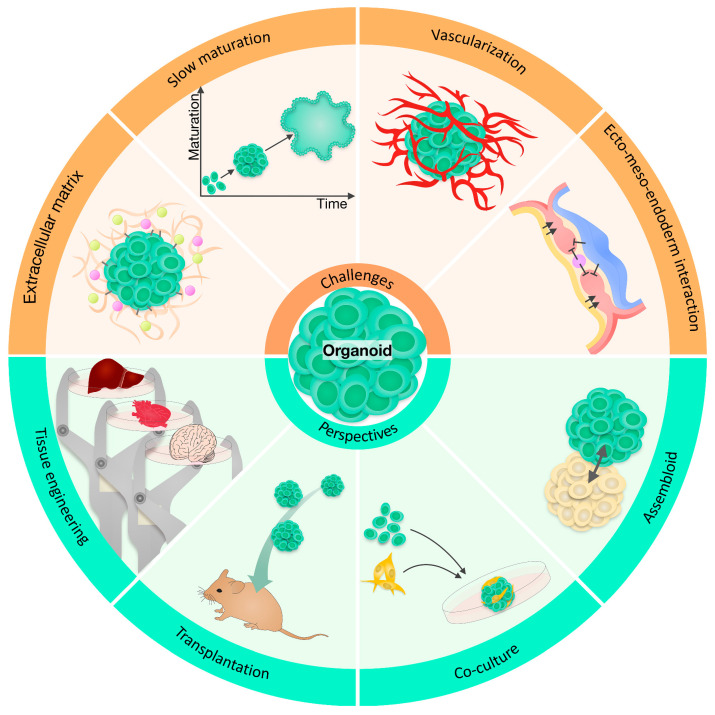
An overview of organoid challenges and perspectives. The schematic illustration represents organoid technology challenges including slow maturation, lack of ECM, vascularization, and ectoderm–mesoderm–endoderm interactions with a foreseeable solution to overcome these weaknesses by transplantation, tissue engineering, co-culture, and assembloids. This figure has been reproduced with permission from [10] granted by the Copyright Clearance Center’s RightsLink® service (License Number: 5455920726198).

Figure 5 presents a general overview of the current challenges and potential of human brain organoids.

## 5. Drug Discovery and Development: Potential and Limitations of Brain Organoids

Drug development for disorders of the central nervous system (CNS) is generally challenging [124]. The challenges can be related to target identification and validation, animal models of diseases, and research infrastructure and resources. The potential for human brain organoids to provide a more dynamic and biological system for drug development and target identification has generated huge interest [125]. Brain organoids can provide the potential for large-scale screening of potential candidates. This advantage can be combined with the application of artificial intelligence and machine learning to accelerate the drug screening development process with a potentially improved success rate. From a long-term perspective, brain organoids might help in reducing the cost of preclinical testing in drug development.

Compared with other organoids that are used for drug screening and development, limited information is available for use of brain organoids for such purposes. However, available literature shows that brain organoids have successfully been used for drug tests in some neurological disorders. For example, in a recent study [126], the neurodegenerative incurable disease of Creutzfeldt–Jakob Disease (CJD) was modeled with the aid of a human cerebral organoid model. This model of human prion disease was used to screen drugs for this disorder, where the researchers tested the effect of pentosan polysulfate (PPS) and identified prion propagation delay and exertion of therapeutic effects [126]. Several other neurodegenerative and neurodevelopmental disorders have been modeled with the aid of organoids [127] such as Alzheimer’s disease (AD), Parkinson’s disease (PD), and autistic spectrum disorders (ASD). These models also offer the potential for drug testing. The development of human CNS barrier-forming organoids (CBFOs) [128] has advanced the potential for testing the permeability of drugs to CNS, hence offering a platform for drug screening in CND drug development. Blood–brain barrier (BBB) organoids could facilitate prescreening of the drug candidates for permeability before the screening of functionality for a potential effect against neurological disorders [127].

Brain organoids have also been employed to screen neurotoxicity in response to various compounds at early developmental stages. Literature shows studies that have identified drugs and heavy metal chemicals with neurotoxicity effects. For example, Liu et al. [129] used cerebral organoids and presented that vincristine could induce dose-dependent neurotoxicity. This model also allowed the researchers to investigate the mechanisms underlying neurotoxicity, where expression of fibronectin, tubulin, and MMP10 was inhibited by vincristine.

Currently, some limitations exist [127] in the application of brain organoids for drug screening and testing that mainly stem from the quantity and quality of organoids in terms of efficiency and reproducibility. For instance, poor quality of organoids resembling the phenotypic characteristics of the original organ or disease can dramatically influence the reliability of results when drugs are tested on these platforms. Another limitation is that while drugs can be tested on brain organoids, their interaction with other organs and systems cannot be tested [127]. Attempts are, however, being reported for overcoming some of these challenges. For example, an integrated system-level approach has been established to determine drug targets for AD and test re-purposing FDA-approved drugs for this disorder [130].

Collectively, these examples trigger the idea that brain organoids might also offer the potential for drug screening in migraine and drug development, such as testing drug effects or toxic effects at the preclinical stage. It is conceivable that iPSCs generated from patients with migraines would provide an experimental platform to test drugs or identify molecular targets, similar to what has been done for other disorders [131]. Migraine is a complex disorder and is influenced by various factors; building up systems that can recapitulate the disease pathogenesis might enable us to accelerate the identification of useful drugs for this disorder. Drug development for migraines is not at the rate or speed of other lines of drug development, for example, drug development for cancer. However, new therapeutics for migraines have also been introduced [132]. After the triptans developed for migraine in the 1990s, a breakthrough came recently with the emergence of ditans, gepants, and anti-calcitonin gene-related peptide monoclonal antibodies against migraines [133].

We must be mindful that organoid models cannot mimic entire migraine pathogenesis, and hence cannot be a perfect model for drug screening either. However, the ambition is to benefit from a collaborative platform provided by advanced technology and research methods and a combination of neuroscience, stem cell biology, neurology, bioengineering, and biomaterials to explore the potential of brain organoids in migraine research and drug screening for this disorder.

## 6. Neuroethical Considerations

Neuroethics [134] cannot be separated from the neuroscience of human brain organoids. Research with ex vivo brain tissue and brain organoids raises several neuroethical questions and concerns, for example, morality and the potential for consciousness [135,136,137]. For instance, the transplantation of organoids in rodent brains has raised some concerns as to how a juncture would be treated as a human–rodent mixture, legally and ethically [137,138]. Recently, a systematic review of ethical issues has been presented [139] to assist in the responsible development and clinical implementation of human brain organoids. A four-step approach has been proposed to assist in overcoming ethical and legal concerns related to the generation and application of organoids [140]. The proposed step consists of the consideration of existing regulations and guidelines with required adjustments, building special regulatory provisions, the involvement of the public, and careful monitoring of the rapid advancements in the neuroscience field to match the speed of consolidation of neuroethics in this field [140].

## 7. Global Cooperation

As human brain organoid projects continue to develop around the world, there remains an ongoing emphasis on the importance of global conversations regarding shared opportunities and challenges, and collaborative international efforts to overcome those challenges to maximize success. Indeed, reproducibility is a challenge in this field, and attempts are ongoing to set standard protocols that are openly shared, discussed, and revised for improvement and harmonization. STAR protocols (Cell Press) are one example of an open-access protocol journal that publishes a wide range of protocols, including human brain organoid protocols. More than 5000 human brain organoid or assembloid protocols have been published as STAR protocols according to GoogleScholar (as of December 2022). A collaborative workplace has recently been created (https://www.protocols.io/ accessed on 3 February 2023) to help achieve collaboration and enhance reproducibility. Within this platform, an area has been dedicated to organoid and assembloid method development, where the community involved in this field can reach out and share information. The protocols are kept organized and up to date.

## 8. Conclusions

To advance the understanding of neural development, and the brain in health and diseases, human brain organoids have been developed. These in vitro models have been generated from pluripotent stem cells that have a unique ability to differentiate into any of the germ layers in vitro, with the aid of 3D cell culture methods. The organoid cultures allow the cells derived from healthy individuals or individuals with brain disorders to self-organize into brain organoids. The cells can also be guided to resemble specific brain regions or to form assembloids. These models are expected to be reliable, realistic, and personalized models of the human brain. To probe these 3D cultures, genetic, anatomical, and functional read-outs are used. Human brain organoid technology combined with bioengineering has opened a wide potential for understanding the human brain and the pathogenesis of neurological and psychiatric disorders. In line with the advancement of brain organoids and assembloids, it is expected that more scalable and improved assays of 3D tissue will be available that can also be used for the identification of therapeutic targets.

Brain organoids are already under investigation and are used for several neurological (e.g., epilepsy, Alzheimer’s disease) and neuropsychiatric disorders (e.g., schizophrenia), and promising results are accumulating rapidly. Migraine is a brain disorder with a hereditary and non-hereditary nature. Migraines are one of the most widespread and disabling non-communicable diseases [64], with a complex pathogenesis and features of biopsychosocial elements [141] that can be considered a potential subject for the application of human brain organoids to further investigate its biological pathogenesis and potential drug development. Here, extending the concept of the possible inclusion and use of brain organoids—among other experimental models used to study migraines [83]—was discussed. The expectation is that by improving the reliability, anatomical accuracy, predictability, and scalability of human brain organoids, this can be achieved. Currently, there is no human brain organoid available for migraines, and this is open for testing. Interested researchers are invited to join the network for protocol development and exchange ideas.

**Figure 6:**
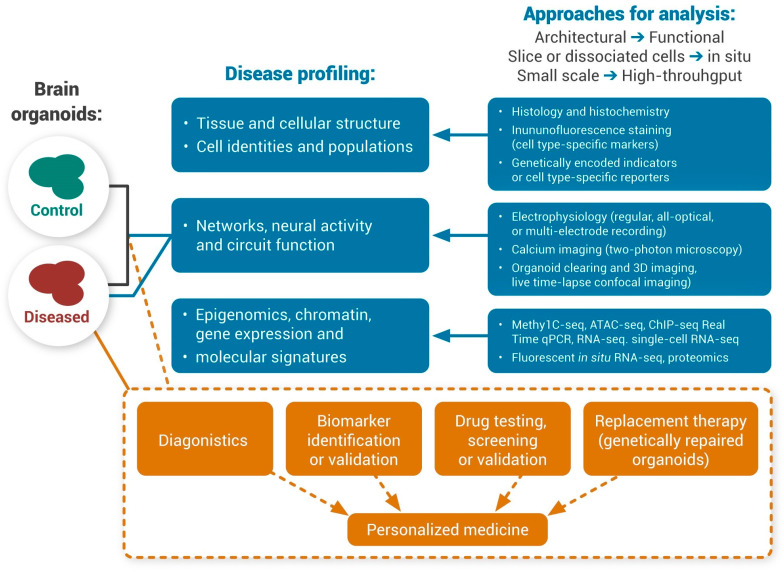
Proposed applications of human brain organoids in neurological diseases. Abbreviations: ATAC-seq, assay for transposase-accessible chromatin with high-throughput sequencing; ChIP-seq, chromatin immunoprecipitation, and sequencing; MethylC-seq, MethylC sequencing; qPCR, quantitative PCR; RNA-seq, RNA sequencing. This figure has been reproduced according to [125] under an open access Creative Common CC BY license granted by Frontiers.

Figure 6 summarizes some of the proposed applications of human brain organoids in neurological diseases [125] that can theoretically be applied to migraine disorder.

It Is expected that modeling brain organoids for migraines will be in line with incorporating various cell types, e.g., glia, endothelial cells, and pericytes, to study neuroimmunological and neurovascular interactions in brain organoids for this disorder. In addition, it is expected that reliable models will provide the capacity to test environmental causes of pathogenesis besides genetic aspects. In the next steps of development, it is proposed that building large-scale platforms for drug discovery and compound screening will also accelerate antimigraine drug development. Currently, the transplantation of organoids into rodents is undergoing rapid development, and this method can also be useful for modeling migraines to obtain circuit-wide integration and advancement of the field.

Careful pairing of the advanced neuroscience of human brain organoids with legal and neuroethical aspects will ensure the ethical generation and application of these complex entities in research and clinical care.

## Data Availability

Not applicable.
